# Case Report: Onset of Takotsubo syndrome during a heart rehabilitation session

**DOI:** 10.3389/fcvm.2025.1560087

**Published:** 2025-06-24

**Authors:** Ana Belén Jiménez-Jiménez, Javier Muñoz-Paz, Diana Ladera-Santos, Ángela Heredia-Torres, Javier Caballero-Villarraso, Fernando Mayordomo-Riera

**Affiliations:** ^1^ GC-28 Group, Maimonides Biomedical Research Institute of Cordoba (IMIBIC), Córdoba, Spain; ^2^ Department of Physical Medicine and Rehabilitation, Reina Sofía University Hospital, Córdoba, Spain; ^3^ Department of Cardiology, Reina Sofía University Hospital, Córdoba, Spain; ^4^ Department of Clinical Analysis, Reina Sofía University Hospital, Córdoba, Spain

**Keywords:** Takotsubo, broken heart syndrome, stress-induced cardiomyopathy, atypical onset, heart rehabilitation

## Abstract

Takotsubo syndrome (TTS) is an acute cardiac condition characterized by transient regional left ventricular systolic dysfunction. Traditionally, it has been associated with physical or psychological stressors. Clinically, the onset of TTS is similar to acute coronary syndrome, requiring appropriate differential diagnosis to distinguish between the two clinical entities. Its treatment is usually conservative, and the prognosis is generally favorable. We report the case of a 57-year-old woman who was referred to the Department of Rehabilitation after suffering a non-ST-segment elevation myocardial infarction. During her eighth physical rehabilitation session, she developed palpitations and hypertension. Initial telemetry monitoring showed ventricular bigeminy with extrasystoles, tachycardia episodes, and ST-segment elevation. She was admitted to the Department of Cardiology, and cardiac catheterization and subsequent coronary angiography were performed, revealing no incidence of coronary lesions. However, ventriculography demonstrated severe left ventricular dysfunction in the systolic phase, characterized by akinesia of the mid-apical segments. These findings were suggestive of Takotsubo syndrome, which was subsequently confirmed by cardiac magnetic resonance imaging.

## Introduction

Takotsubo syndrome (TTS), also known as “broken heart syndrome” or “stress-induced cardiomyopathy,” is a rare clinical entity. Its prevalence is estimated to be 1%–2% in patients presenting with suspected acute coronary syndrome (ACS) (1). Keeping in mind certain contexts in which this disease can appear is essential to facilitate early and accurate diagnosis and ensure proper treatment.

Takotsubo syndrome is a nonischemic heart disease that usually affects postmenopausal women. It has traditionally been associated with emotional or physical stressors, such as an intense emotional reaction, typically occurring within 5 days prior to symptom onset. Although the etiological mechanisms remain unknown, it is believed that sympathetic discharge plays a key role. This sympathetic discharge can lead to myocardial contraction, microvascular dysfunction, myocardial infarction, and systolic dysfunction (2).

Its clinical manifestations and electrocardiogram abnormalities often mimic those of ACS, especially in the early phase. Often reported symptoms are chest discomfort, electrocardiogram abnormalities such as ST-segment elevation or depression, and cardiac rhythm abnormalities on ECG. In some cases, however, the ECG may appear normal. Blood tests usually show high serum levels of troponin. Therefore, an accurate differential diagnosis is important between the two diseases (3, 4). Nevertheless, TTS manifests as wall motion abnormalities in the absence of obstructive coronary artery disease, as confirmed by angiography.

The typical pattern of abnormal regional left ventricular motion in TTS includes apical hypokinesia, akinesia, or dyskinesia with apical ballooning, accompanied by relative basal hyperkinesia, resembling the shape of octopus traps used in Japan. Atypical forms of TTS also exist, which are characterized by mid-ventricular or basal hypokinesia. Focal stress-induced TTS is another rare form that closely mimics myocarditis or myocardial infarction, making differential diagnosis challenging. In particular, midventricular TTS is characterized by motion abnormalities in the middle portion of the left ventricle, while the apical region remains normokinetic or hyperkinetic. Atypical TTS presents with a peculiarity: ECG abnormalities often include ST-segment depression and less profound T-wave inversion in leads I and aVL. Patients with atypical TTS are usually younger than those with typical TTS, and there are usually no differences in symptoms or emotional or physical triggers (5).

Cardiac magnetic resonance (CMR) is a valuable, non-invasive diagnostic tool that facilitates the differential diagnosis between ischemic and nonischemic etiologies (acute myocarditis, Takotsubo syndrome, and other conditions) of myocardial injury (6).

TTS usually responds well to conservative treatment, with complete recovery of systolic function after several weeks or months. However, sometimes, heart rehabilitation programs may be required. Even so, its prognosis is generally favorable (7, 8).

## Case description

The patient is a 57-year-old woman with a medical history of dyslipidemia managed with statins and a history of smoking (since 2 years ago) and appendectomy. She was receiving treatment with omeprazole, aspirin, ticagrelor, bisoprolol, ramipril, atorvastatin/ezetimibe, and eplerenone because of a non-ST-segment elevation myocardial infarction (NSTEMI) in June 2022.

She was referred to the Department of Rehabilitation to begin heart physical therapy 7 months after suffering an NSTEMI. The NSTEMI was caused by a total obstruction in the proximal to mid-segment of the right coronary artery, and it was treated with revascularization using a Biofreedom stent (2.5 × 22 mm). The patient had a mildly depressed left ventricular ejection fraction (LVEF 50%) because of septal and inferior hypokinesia and presented with symptoms of acute heart failure, including dyspnea, which was classified as NYHA functional class II, and orthopnea.

Before beginning rehabilitation treatment, an initial evaluation was carried out, which consisted of an ergospirometry test performed on a treadmill following an incremental Naughton ramp protocol. The test was terminated due to patient exhaustion; however, it was clinically and electrocardiographically negative for ischemic heart disease. The patient passed the test and was included in a medium cardiac risk group (score 0+).

After 5 months of starting the rehabilitation program, the patient told us of an intense emotional reaction related to a family problem that occurred 2 days before the eighth rehabilitation session. The patient said that she felt worried. During physical training, her heart rate and rhythm were monitored by electrode patches placed on her chest. The telemetry data on heart rate and rhythm were projected onto a medical computer and supervised by a doctor, allowing observation of changes in cardiac rate or rhythm while the patient walked on the treadmill.

**Figure 1:**
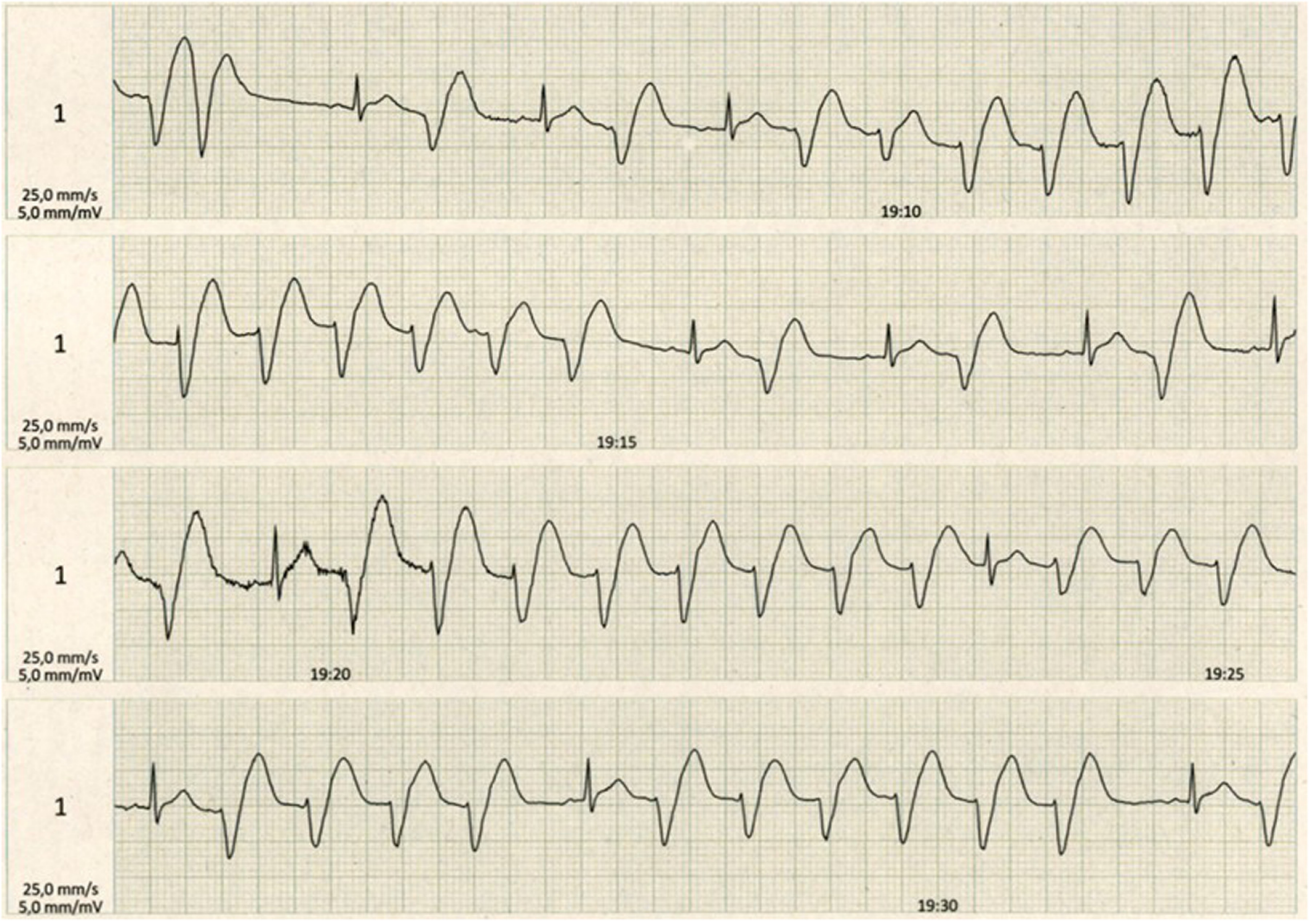
Telemetry during exertion in the eighth session of the heart rehabilitation program showing ventricular bigeminy with extrasystole and tachycardia with wide QRS complex episodes reaching 130–140 beats per minute and ST-segment elevation.

During the eighth rehabilitation session, the patient walked on the treadmill at 4.5 km/h with no incline. She began experiencing palpitations, but she did not report dyspnea, thoracic pain, or fainting. Telemetry showed ventricular bigeminy, which is characterized by extrasystole and tachycardia episodes reaching 130–140 beats per minute (bpm), a wide QRS complex during exertion, and ST-segment elevation (Figure 1). As a result of this episode, the training session was halted. Arterial pressure was measured, and it indicated 180 mmHg (diastolic) and 120 mmHg (systolic). During the acute phase, a blood test revealed a serum level of high-sensitivity troponin of 3.012 ng/L (normal range: <0.4 ng/L). However, the cardiac rhythm and arterial pressure normalized after 30 min of rest.

**Figure 2:**
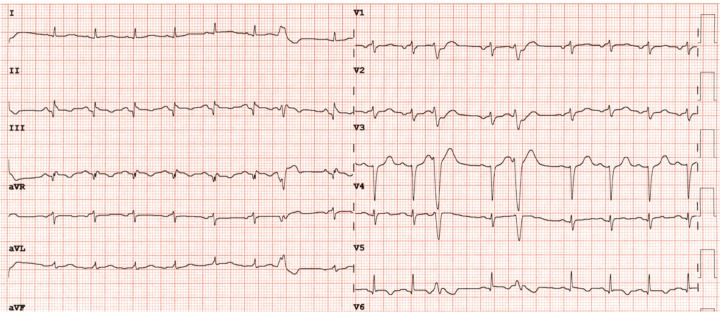
ECG performed in the Cardiology Department demonstrating sinus tachycardia at 100 bpm, narrow QRS complexes, inferior Q waves, slow R-to-S progression in the precordial leads, and isolated J-point elevation in lead V6.

**Figure 3:**
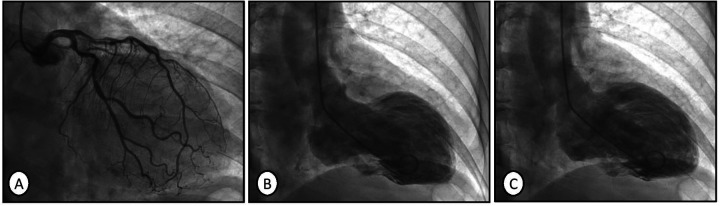
Coronary angiography with no evidence of coronary artery obstruction **(A)**. Ventriculography performed in the left ventricle revealing akinesia and ballooning of the middle and apical segments , indicating severe dysfunction in the end-systolic phase **(B)**. Normal left ventricular contraction during the end-diastolic phase **(C)**.

The patient was admitted to the Department of Cardiology. A beta-blocker dose was prescribed, and the heart rhythm was monitored. Another ECG performed after 3 h demonstrated sinus tachycardia at 100 bpm, a narrow QRS complex with inferior Q waves, slow R-to-S progression in the precordial leads, and isolated J-point elevation in lead V6 (Figure 2). The patient was then transferred to the hemodynamics unit, where catheterization and subsequent coronary angiography were performed, which did not reveal coronary artery obstruction (Figure 3A). Thus, ischemic heart disease was ruled out. Afterward, left ventriculography demonstrated akinesia and ballooning of the middle and apical segments and severe systolic dysfunction (Figure 3B), with no left ventricular contraction during the diastolic phase (Figure 3C). These findings suggested a diagnosis of TTS.

**Figure 4:**
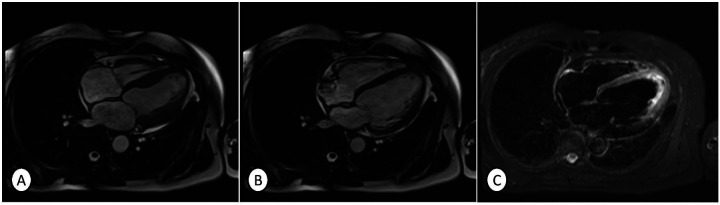
Cardiac magnetic resonance imaging showing a dilated, non-hypertrophic left ventricle with a moderately depressed left ventricular ejection fraction (LVEF 39%) and noticeable hypokinesia in the mid-apical segments during systole **(A)** and normal contraction in the diastole **(B)**, and T2-STIR sequence showing a marked increase in signal intensity in the mid-apical segments, consistent with diffuse myocardial edema **(C)**. No intracavitary thrombi or pericardial or pleural effusions were observed. Therefore, according to these findings, the patient was diagnosed with TTS.

During this time, the patient remained asymptomatic, except for an increase in cardiac frequency during minimal exertion, but these symptoms resolved gradually. On day 4, a CMR scan was performed, and it confirmed the diagnosis of TTS. Imaging showed a dilated, non-hypertrophic left ventricle with a moderately reduced left ventricular ejection fraction (LVEF 39%) and noticeable hypokinesia of the mid-apical segments during systole (Figure 4A), while diastolic function remained normal (Figure 4B). Additionally, the T2-STIR sequence showed a marked increase in signal intensity in the mid-apical segments, consistent with diffuse myocardial edema (Figure 4C). In addition, a previous infarction area appeared in the right coronary artery territory. No intracavitary thrombi, pericardial effusion, or pleural effusion were observed. Therefore, according to these findings, the patient was diagnosed with TTS.

Discharge recommendations included maintaining a healthy lifestyle, following the Mediterranean Diet, avoiding stressful situations and potential emotional triggers, controlling cardiovascular risk factors under the care of a primary attention physician, recognizing warning signs requiring evaluation at the Emergency Department, and continuing treatment with acetylsalicylic acid, an angiotensin-converting enzyme inhibitor, a beta-blocker, and statins.

The clinical course was uncomplicated, and the patient was included again in a heart rehabilitation program to improve her ventricular function. She completed the program without complications. After 5 months of completing the rehabilitation program, a planned transthoracic echocardiogram indicated complete recovery of left ventricular function, with normalization of global and segmental systolic function and a preserved left ventricular ejection fraction (LVEF 55%).

## Discussion

TTS is a rare heart disease characterized by transient left ventricular systolic dysfunction, which usually affects the apex. The primary differential diagnosis is ACS due to similar electrical, biochemical, and clinical presentations (9). As we mentioned in the diagnosis of the reported patient, the acute phase of TTS often involves ECG abnormalities (cardiac arrhythmias, ST-segment elevation, or Q waves), clinical symptoms, and high troponin levels in blood tests—features commonly seen in both diseases, complicating the differential diagnosis (10). However, coronary angiography, ventriculography, and cardiac magnetic resonance imaging support a TTS diagnosis.

For this reason, patients undergoing a heart rehabilitation program should be monitored effectively to enable the early detection of ACS (the most frequent complication), as well as other rare diseases such as TTS.

The etiology of TTS remains unclear, although it has been reported to be associated with emotional or physical stress (11, 12). Other cases of TTS triggered by stressful physical situations (such as hypoglycemia or a traumatic amputation) have also been reported (13, 14). Although TTS is usually associated with negative emotional events (such as fear or anxiety), it can also occur in spectators experiencing intense positive emotions during stressful sporting events (15).

It has been proposed that these stressful factors are related to sympathetic discharge and elevated catecholamine levels (16). This can lead to myocardial stunning, microvascular dysfunction, myocardial microinfarction, and, consequently, typical left ventricular systolic dysfunction (4). Recent evidence suggests that TTS may be a form of ACS due to microvascular dysfunction (17, 18). In this regard, a case of TTS has been reported in a patient after a COVID-19 infection (19, 20).

This case has two peculiarities: (i) TTS onset occurred in a cardiac monothyrization setting during a training session (in the Department of Rehabilitation), which allowed for an early diagnosis; and (ii) two etiological factors were present at the same time. It should be considered that ventricular tachycardia appearance after physical training and previous emotionally stressful events could have a synergistic effect on the myocardium. These actions (especially when combined) could result in a potential adrenergic discharge and, therefore, a greater probability of the development of left ventricular dysfunction.

As with this patient, TTS treatment is usually conservative, involving expectant behavior and monitoring for arterial pressure and the possible appearance of cardiac arrhythmias. This dysfunction is generally transient, and the majority of patients recover systolic function within weeks to months. Occasionally, they require inclusion in heart rehabilitation programs (21, 22).

From the patient's perspective, she told us that verbalizing this family problem helped her feel more at peace. Furthermore, the continuous care by the nurses (monitoring blood pressure and heart rhythm) made her feel cared for and looked after, and she demonstrated a grateful attitude every time.

## Conclusion

It is well-known that the risk of cardiovascular complications increases after an ACS, particularly within the first 5 years. There is a greater probability of developing TTS in the first 4 weeks after ACS (23). Despite the possibility of this heart complication, we found no other cases in the scientific literature reporting the onset of TTS in patients enrolled in a heart rehabilitation program after an ACS, particularly during therapy sessions. For this reason, we would like to emphasize the rigor of the established guidelines, algorithms, and the importance of bearing in mind this rare condition, even in unusual contexts. Undoubtedly, these diagnostic algorithms lead to an appropriate syndromic classification because they involve a correct exclusion diagnosis of ACS.

## Data Availability

The original contributions presented in the study are included in the article/**Supplementary Material**; further inquiries can be directed to the corresponding author.
