# Supplementary material for: Case Report: Onset of Takotsubo syndrome during a heart rehabilitation session
Source: Front Cardiovasc Med. 2025 Jun 24;12:1560087. doi: 10.3389/fcvm.2025.1560087 (PMC12234314; doi:10.3389/fcvm.2025.1560087)
Supplement: Supplementary file 1 [file Supplementaryfile1.docx]

Supplementary Material

# Supplementary Data

Supplementary Material should be uploaded separately on submission. Please include any supplementary data, figures and/or tables.

Supplementary material is not typeset so please ensure that all information is clearly presented, the appropriate caption is included in the file and not in the manuscript, and that the style conforms to the rest of the article.

# Supplementary Figures and Tables

For more information on Supplementary Material and for details on the different file types accepted, please see [here](https://www.frontiersin.org/guidelines/author-guidelines#supplementary-material).

| **Table 1. Timeline.** | |
| --- | --- |
| **Sequence of events** | |
| **Possible trigger** | Emotional stressful event (familiar problem) and physical stress during eighth session of heart rehabilitation. |
| **Clinical presentation** | Palpitations and high blood pressure. |
| **EKG in heart rehabilitation session** | Bigeminism, ventricular extrasystole and tachycardia, ST elevation. |
| **Laboratory** | Increase of high-sensitive troponin level. |
| **Cardiology department** | Monitoring cardiac rhythm and second EKG with sinus tachycardia, narrow QRS complex, inferior Q waves and slow RS progression in precordial leads. |
| **Cardiac catheterization** | **Angiography:** No coronary obstruction.  **Ventriculography**: Left ventricular apical akinesia and severe dysfunction. |
| **CMR** | Hypokinesia in middle-apical segments during systole, depressed ejection fraction and diffuse myocardial edema confirm TTS diagnosis. |
| **Outcome** | Asymptomatic, left ventricle complete functional recovery in echocardiogram and reincluded in heart rehabilitation program without new incidents. |

## Supplementary Figures


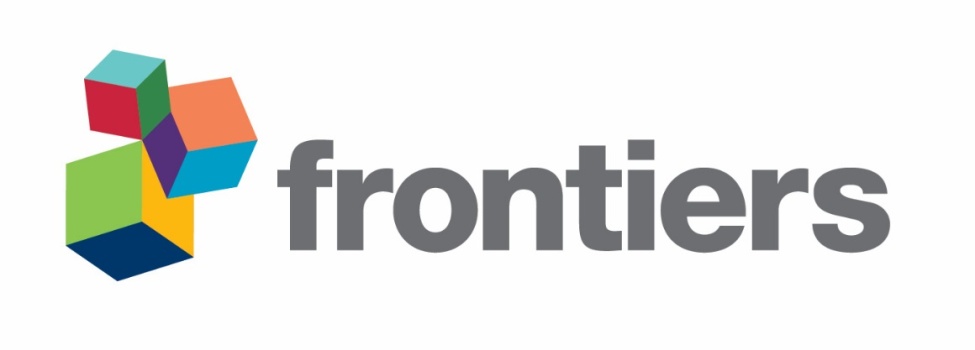


**Supplementary Figure 1.** Telemetry during effort in eighth session of heart rehabilitation evidenced ventricular bigeminism with extrasystole and tachycardia with wide QRS complex attacks of 130-140 beats per minute and ST elevation.

**Supplementary Figure 2.** EKG done in Cardiology Department demonstrated sinus tachycardia at 100 bpm, narrow QRS complex, inferior Q waves and slow RS progression in precordial leads with isolated J point elevation in V6.

**Supplementary Figure 3.** Coronary angiography which did not document coronary artery obstruction (**A**). Ventriculography performed in left ventricle reflected akinesia and ballooning of middle and apical segments and severe dysfunction on end-systole phase (**B**). Left ventricle contraction normality during end diastole phase (**C**).

**Supplementary Figure 4.** Cardiac magnetic resonance showed a dilated, non-hypertrophic left ventricle with a moderately depressed left ventricular ejection fraction (LVEF 39%) with a noticeable hypokinesia in middle-apical segments during systole (**A**) with normality in diastole (**B**) and T2-Stir sequence shows a marked increase in signal intensity in the mid-apical segments, consistent with diffuse myocardial edema (**C**). No intracavitary thrombi or pericardial or pleural effusion were observed. Therefore, according to these findings she was diagnosed with TTS.
